# Development of a novel bi-specific monoclonal antibody approach for tumour targeting

**DOI:** 10.1038/sj.bjc.6690712

**Published:** 1999-10

**Authors:** A A Koumarianou, M Hudson, R Williams, A A Epenetos, G W H Stamp

**Affiliations:** Department of Histopathology, Imperial College School of Medicine, Hammersmith Campus, Du Cane Road, London, W12 0NN, UK; Antisoma, St Georges Hospital, Cranmer Terrace, London, SW17 0QS, UK

**Keywords:** bi-specific monoclonal antibody, tumour immunotherapy, directly streptavidinylated antibody, biotinylated anti-CD3 Fab fragments, MUC1 mucin

## Abstract

To overcome the disadvantages of bi-specific antibody methodologies in vivo, a novel antibody approach has been designed to improve tumour targeting and effector to target ratio. The technique involves biotinylated anti-CD3 Fab fragments and streptavidinylated anti-tumour monoclonal antibodies (mAbs) that can spontaneously form cross-links. We describe here a method for the direct cross-linking of sulphydryl-conjugated HMFG1 (anti-MUC1 mucin mAb) to streptavidin by sulphosuccinimidyl-4-(*N*-maleimidomethyl) cyclohexane- 1-carboxylate. Fab fragments generated by papain digestion of the 1452C11 antibody (anti-CD3 mAb without Fc to avoid peripheral activation of T-cells) were biotinylated with NHS-Iminobiotin. MUC1-transfected BALB/c breast cancer cell lines 413BCR and 425CCR and the parental cell line (410.4) were labelled with streptavidinylated mouse anti-MUC1 mucin mAb. BALB/c effector T-cells were separately labelled with biotinylated anti-CD3 Fab fragments (1452C11) and mixed with tumour cells in different effector to target ratios. Percentage of killing was assessed using the ^51^Cr cytotoxicity assay. Seventy per cent lysis was measured in the case of 413BCR (high MUC1 mucin expressor) and 40% in the case of 425CCR (low expressor) cell line. No lysis was apparent in the MUC1 negative cell line. These results demonstrate that the novel T-cell redirecting approach we have developed can produce effective immune lysis of target cells in vitro. © 1999 Cancer Research Campaign


*British Journal of Cancer* (1999) **81**(3), 431–439
© 1999 Cancer Research Campaign
Article no. bjoc.1999.0712

### Development of a novel bi-specific monoclonal antibody approach for tumour targeting

**AA Koumarianou[1], M Hudson[1], R Williams[1], AA Epenetos[2] and GWH Stamp[1]**

[1]Department of Histopathology, Imperial College School of Medicine, Hammersmith Campus, Du Cane Road London W12 0NN, UK; [2]Antisoma, St Georges Hospital, Cranmer Terrace, London SW17 0QS, UK

**Summary** To overcome the disadvantages of bi-specific antibody methodologies in vivo, a novel antibody approach has been designed to improve tumour targeting and effector to target ratio. The technique involves biotinylated anti-CD3 Fab fragments and streptavidinylated anti-tumour monoclonal antibodies (mAbs) that can spontaneously form cross-links. We describe here a method for the direct cross-linking of sulphydryl-conjugated HMFG1 (anti-MUC1 mucin mAb) to streptavidin by sulphosuccinimidyl-4-(*N*-maleimidomethyl) cyclohexane-1-carboxylate. Fab fragments generated by papain digestion of the 1452C11 antibody (anti-CD3 mAb without Fc to avoid peripheral activation of T-cells) were biotinylated with NHS-Iminobiotin. MUC1-transfected BALB/c breast cancer cell lines 413BCR and 425CCR and the parental cell line (410.4) were labelled with streptavidinylated mouse anti-MUC1 mucin mAb. BALB/c effector T-cells were separately labelled with biotinylated anti-CD3 Fab fragments (1452C11) and mixed with tumour cells in different effector to target ratios. Percentage of killing was assessed using the $^{51}$Cr cytotoxicity assay. Seventy per cent lysis was measured in the case of 413BCR (high MUC1 mucin expressor) and 40% in the case of 425CCR (low expressor) cell line. No lysis was apparent in the MUC1 negative cell line. These results demonstrate that the novel T-cell redirecting approach we have developed can produce effective immune lysis of target cells in vitro. © 1999 Cancer Research Campaign

**Keywords**: bi-specific monoclonal antibody; tumour immunotherapy; directly streptavidinylated antibody; biotinylated anti-CD3 Fab fragments; MUC1 mucin

Tumour targeting by means of monoclonal antibodies (mAbs) directly conjugated to isotope or drugs has been extensively investigated for imaging and therapy with relatively disappointing results.

Alternative methods for tumour targeting include the indirect (two-step) delivery system where a reagent can interact specifically with both the target and effector compound. This system involves the use of bi-specific antibodies (BiAbs) which have two different specific antigen binding sites, one for the tumour-associated antigen and one for the effector compound (e.g. the CD3 receptor of T-cells or an isotope such as $^{111}$In). BiAbs that cross-link the T-cell-associated TcR/CD3 receptor with the tumour cells, promote an activation signal that mediates cytotoxicity against the target cell in an MHC-independent way, regardless of the T-cell specificity (Perez et al, 1985; Staerz et al, 1985). In very few studies such as a lymphoma model in mice (Demanet et al, 1992) and a small clinical trial of intracavitary administration in glioma patients (Nitta et al, 1990) cure of established tumours was achieved. Furthermore, BiAb application in vivo has not been shown to result in specific localization of BiAbs and T-cells in solid tumours.

A more sophisticated version of the indirect approach for tumour targeting is offered by the 'universal bispecific antibody approach' (Gilliland et al, 1988). In this system, specific anti-tumour mAbs are first localized at the target site. The universal BiAb, an anti-CD3 × anti-Ig BiAb, is then administered. The anti-Ig arm binds to the specific anti-tumour mAbs and the anti-CD3 arm cross-links the CD3 receptor to activate cytotoxic T-cells, leading to the destruction of tumour cells. A similar approach has been used to target recombinant Ab fragments (George et al, 1994). This approach has the advantage of being less expensive since the 'universal BiAb' can be used to redirect effector cells against a variety of tumours, and offers the opportunity of targeting more than one tumour associated antigen at the same time (George et al, 1994).

The indirect approach has also been modified for in vivo tumour imaging studies providing data on pharmacokinetics and biodistribution. A first step injection of anti-tumour × anti-tracer BiAb is followed by injection of the tracer compound (Le Doussal et al, 1992). The advantages of the indirect in vivo imaging compared to the direct approach (mAb directly conjugated to tracer) include: (i) higher tumour/tissue ratios (especially with liver, spleen and kidneys) and (ii) faster contrast images.

An alternative approach for signal amplification in tumour targeting is represented by the streptavidin (SA)–biotin indirect system for tumour imaging which has resulted in the further reduction of background noise and amplification of the target bound signal (Hnatowich et al, 1987; Paganelli et al, 1988; Kalofonos et al, 1990; Zhu et al, 1998). The technique which exploits the high affinity of biotin for SA, consists of an initial injection of streptavidin-conjugated antibodies that are accumulated at the target sites. After blood clearance of free SA–mAb, radiolabelled biotin is injected that binds to the target-bound SA–mAbs while the remaining free radiobiotin clears rapidly from the blood by glomerular filtration. Zhu and colleagues demonstrated a 200%

*Received 3 December 1998*
*Revised 22 April 1999*
*Accepted 22 April 1999*

*Correspondence to:* GWH Stamp

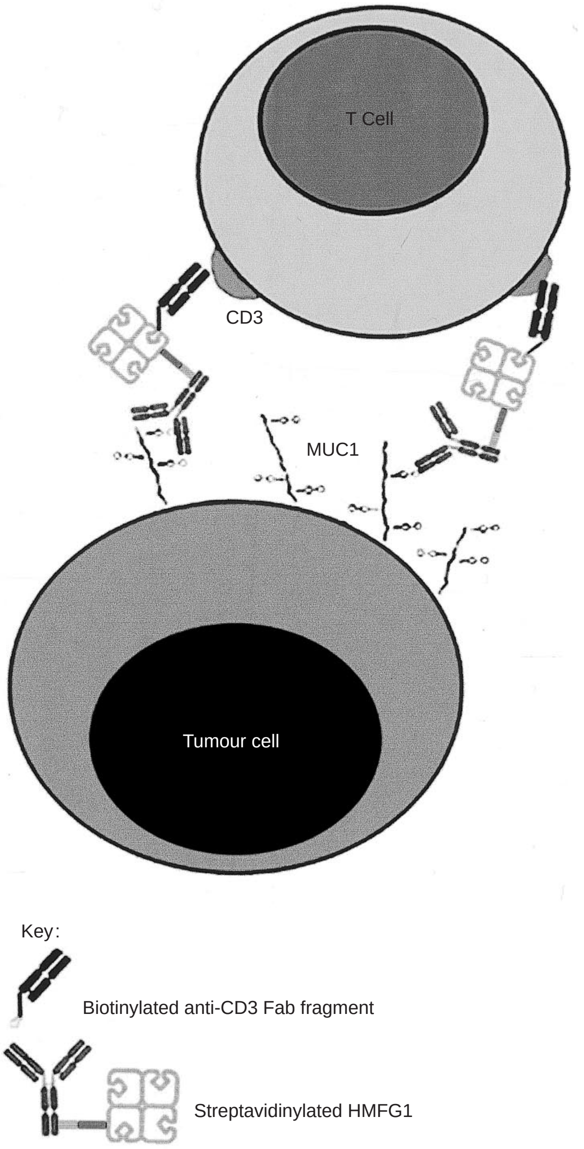

**Figure 1** The strategy of the novel antibody approach for the redirection of T-cells against tumour cells. MUC1 mucin-expressing tumour cells labelled with streptavidinylated anti-MUC1 MAb (HMFG1 SA) cross-linking biotinylated anti-CD3 Fab fragments (Fab Bio) bound on ConA/IL-2 preactivated T-cells by biotin–streptavidin bond increase in the tumour:background ratio with the SA–biotin approach as compared with the BiAb method (Zhu et al, 1998). Furthermore, the latter study showed that tumour antigen expression and shedding had little effect on the isotope delivery to the tumour. Despite the potential benefits of this approach, it has yet to be investigated in the therapeutic context.

The aim of this study has been to develop the SA–biotin approach to improve T-cell redirection at the tumour site for immunotherapeutic applications. In order to achieve this we have directly streptavidinylated an anti-tumour antibody and biotinylated monovalent antibody Fab fragments (without Fc region to avoid T-cell activation/sequestration by cells expressing membrane receptors for the Fc) raised against the CD3 receptor on T-cells. In theory, tumour cells targeted by the antibody conjugated with SA will bind the biotinylated anti-CD3 Fab causing its aggregation (i.e. making an artificially bivalent or polyvalent antibody construct), thus rendering it able to deliver an efficient activation signal for the T-cells (Figure 1). We selected MUC1 mucin, a well-documented antigen of human adenocarcinomas (Taylor et al, 1994) as the target tumour antigen.

### MATERIALS AND METHODS

### Target BALB/c breast cancer cell lines

Five cell lines were used in this study: the mouse 410.4 parental cell line, three transfected clones (435DNV, 413BCR, 425CCR) and the human MCF-7 cell line.

Tumour cell lines used as target originated from 410.4, the 4th transplant generation (Blazar et al, 1980) of a lung metastatic nodule (410) isolated from the 10th subcutaneous passage of an original mammary tumour that arose spontaneously in a strain BALB/c breeding female (Heppner et al, 1978). The 410.4 cell line has a high malignant potential, metastasizes spontaneously with high frequency (> 80%) to the lungs of mice injected subcutaneously or intravenously, and shows lack of immunogenicity (Miller et al, 1993). The 410.4 cell line was transfected with 3.9 kb full-length MUC1 cDNA, kindly provided by Dr M Hollingsworth (Batra et al, 1991), under the control of the β-actin promoter, in the mammalian expression vector pHb-Apr-1-neo by the calcium phosphate precipitation method (M Hudson et al, manuscript in preparation). Transfected clones were selected in Dulbecco's modified Eagle's medium containing 600 μg $ml^{-1}$ G418-sulphate, 10% fetal bovine serum (FBS), 100 U $ml^{-1}$ penicillin, 100 μg $ml^{-1}$ streptomycin and 0.2 μg $ml^{-1}$ butyl-p-hydroxybenzoate (all from Gibco-BRL). The parental line was grown in a similar media but without G418-sulphate.

In this study three transfected cell lines were used: the 435DNV (G418 resistant; vector control) and the MUC1 mucin-transfected clones 413BCR and 425CCR.

The MCF-7, a human breast adenocarcinoma cell line, isolated from a patient's pleural effusion (Soule et al, 1973) and previously shown to express MUC1 mucin (Devine and McKenzie, 1992), was used as a positive control cell line.

### T-cell purification and culture of BALB/c effector T-cells

T-cells were purified from spleens of 6–10 weeks old female BALB/c mice using a mouse T-cell purification column according to the manufacturer's instructions (Pierce). Samples were analysed by flow cytometry for CD3 epitope density using the 500A2 mAb (Havran and Allison, 1988). Preactivated cytotoxic T lymphocytes (CTL) were generated in bulk culture using media containing RPMI-1640 (Gibco), 10% heat inactivated FBS (Life Technologies) pre-screened to support T-cell growth, 100 U $ml^{-1}$ penicillin, 100 μg $ml^{-1}$ streptomycin, 2 mM L-glutamine, $5 \times 10^{-5}$ M 2-mercaptoethanol, 4 μg $ml^{-1}$ Concavalin A (ConA; Sigma) and 50 U $ml^{-1}$ human recombinant interleukin 2 (IL-2 endotoxin tested; Boehringer Mannheim), and maintained in a 5% carbon dioxide humidified incubator, at 37°C. T-cell proliferation was tested by flow cytometry using propidium iodide for DNA staining as previously described (Nicoletti et al, 1991).

 © 1999 Cancer Research Campaign

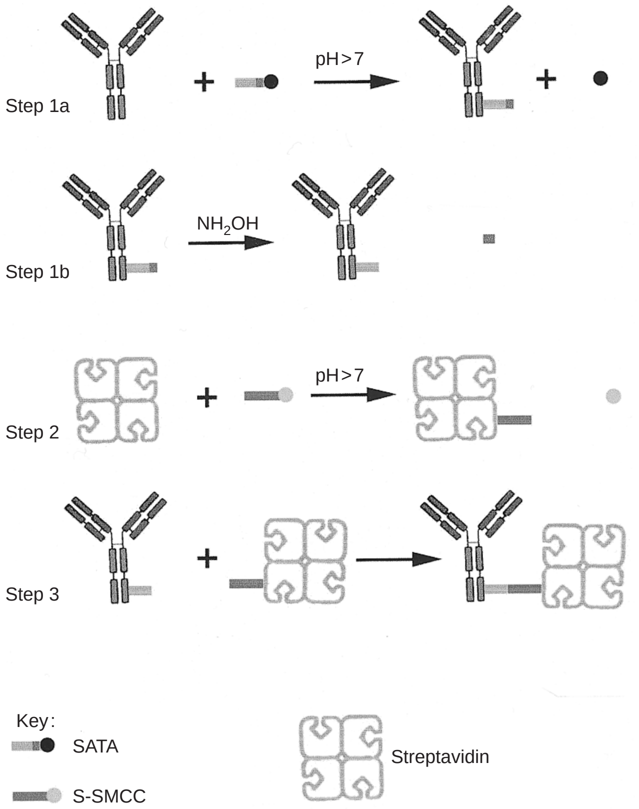

**Figure 2** Schematic representation of the reactions involved in the direct streptavidinylation of HMFG1 mAb. Step 1: (a) Introduction of a -SH group, protected from oxidation by an acetyl group, on the HMFG1 mAb by employing SATA. (b) Deprotection of -SH group by removing the acetyl group with hydroxylamine ($NH_2OH$). Step 2: Addition of a maleimide group onto streptavidin using S-SMCC. Step 3: Streptavidinylation of HMFG1 mAb by a covalent amide-thioether bond

### The two-step targeting approach

### The tumour targeting step

***Anti-MUC1 mucin monoclonal antibody.*** HMFG1, an IgG1 mAb raised in mouse against the human MUC1 mucin was kindly donated by the Imperial College Research Fund (ICRF; London, UK). This antibody that recognizes the PDTR sequence of the mucin's tandem repeat protein backbone was previously shown to specifically localize to malignancies expressing MUC1 mucin in the context of imaging studies (Epenetos et al, 1982). Cell binding studies by flow cytometry were carried out to test HMFG1 binding to MUC1 mucin expressing cells using as second layer fluorescein isothiocyanate (FITC)-conjugated rabbit anti-mouse serum (Dako).

***Streptavidinylation of anti-MUC1 mucin HMFG1 mAb.*** Streptavidinylation of the HMFG1 IgG mAb was achieved by three conjugation reactions described in Figure 2. In the first reaction N-hydroxysuccinimide ester of S-acetylthioacetic acid (SATA; Pierce) was used to introduce a free sulphydryl (-SH) group onto HMFG1 as previously described (Duncan et al, 1983). SATA:IgG ratios were assayed in the range of 1:1 to 20:1 and -SH incorporation was tested using 5,5′-dithio-bis-(2-nitrobenzoic acid) (Pierce) as previously described (Ellman, 1959). In the second reaction, sulphosuccinimidyl 4-[N-maleimidomethyl] cyclohexane-1-carboxylate (S-SMCC; Pierce) was used to introduce a maleimide group onto SA (Pierce) according to a method previously described (Hashida et al, 1984). In the third reaction the two conjugates were mixed together in equimolar ratios to react by a sulphydryl-maleimide bond. Streptavidinylated HMFG1 was separated from unreacted streptavidin and antibody aggregates using microconcentrators of appropriate molecular weight cut-off (Amersham). Successful HMFG1 streptavidinylation was tested by binding studies to 413BCR cells using flow cytometry and cytospin preparations. Negative control experiments included 410.4 parental cell line and 413BCR transfectant cells incubated with cross-linking reagents, SA or anti-SA serum.

### The T-cell redirection step

***Anti-CD3 mAb.*** 1452C11, an Armenian hamster IgG mAb reacts with the 25-kDa ε chain of the T-cell receptor-associated CD3 complex and can activate primed and unprimed T-cells in vitro and in vivo (Leo et al, 1987; Portoles et al, 1989). 1452C11 mAb can also induce redirected lysis of Fc receptor-bearing target cells by CTL clones (Leo et al, 1987). 1452C11 was acquired in no azide/low endotoxin format from PharMingen UK. Fab fragments were generated by papain digestion (Pierce) and purified from Fc and intact IgG using standard affinity chromatography procedures with Protein G 4FF column (Pharmacia). Non-reducing 12.5% sodium dodecyl sulphate-polyacrylamide gel electrophoresis (SDS-PAGE) (Laemmli et al, 1970) and staining with Coomassie brilliant blue were carried out to monitor Fab fragmentation and purification.

***Biotinylation of anti-CD3 Fab fragments.*** Biotinylation of Fab fragments was carried out using N-hydroxysuccimidoiminobiotin hydrobromide (Pierce) at different molar ratios (Biotin/Fab 7:1–50:1) as previously described (Orr, 1981). Fab fragments were tested for biotin content using the HABA method ([2-(4′-hydroxyazobenzene) benzoic acid]; Pierce) as previously described (Green, 1965). Biotinylated anti-CD3 Fab fragments were separated from unreacted biotin and antibody aggregates using microconcentrators of appropriate molecular weight cut-off. In addition, biotinylated Fab fragments were tested for specificity and ability to bind SA by flow cytometry and cytospin preparations of T-cells using as detecting reagent FITC-conjugated SA (Dako).

### Testing the two-step approach

### Flow cytometry

Single-cell suspension of tumour cell lines or T-cells were resuspended in 2% FBS/PBS. Then $10^6$ cells were incubated with first layer antibody (10 μg $ml^{-1}$) for 30 min. Following washes in 2% FBS/PBS to remove unbound antibody, cells were incubated with second layer antibody (same procedure was applied for more antibody layers) for 20 min at 4°C in the dark, and three washes were performed after every layer to remove unbound reagent. After a final incubation in FITC-labelled antibody solution, cells were washed and resuspended in 2% FBS/PBS and subsequently analysed in a Coulter Epics XL-MCL cell sorter for fluorescence emission at 525 ± 10 nm (Fl1). The cross-linking property of the streptavidinylated anti-tumour mAb and biotinylated anti-CD3 Fab fragments was also tested by flow cytometry. Briefly, T-cells were incubated initially with excess biotinylated anti-CD3 Fab fragments then with excess streptavidinylated HMFG1 mAb followed by rabbit anti-SA serum (Sigma) and then by FITC-conjugated goat anti-rabbit serum (Dako) at the concentrations indicated by the manufacturers. Negative control experiments

© 1999 Cancer Research Campaign

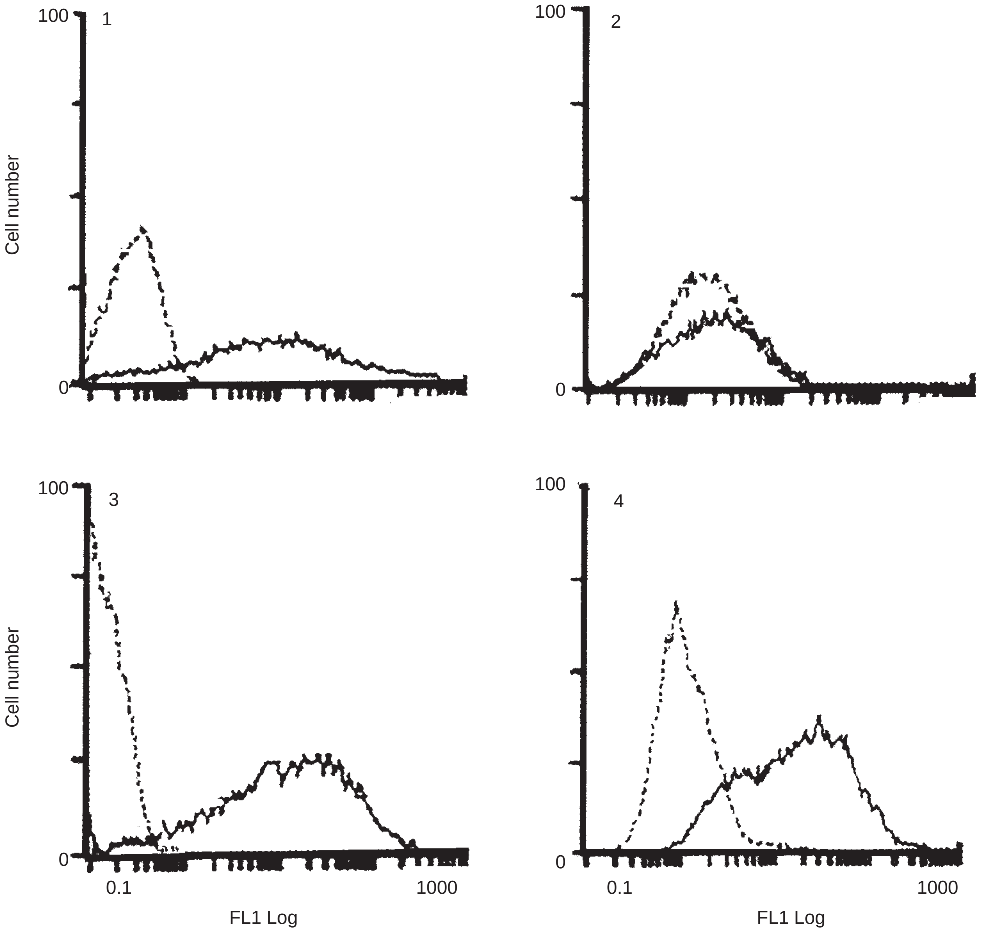

**Figure 3** Cell binding studies of HMFG1 MAb by flow cytometry. Testing of HMFG1 mAb binding to (1) 413BCR; (2) 410.4; (3) MCF-7; (4) 425CCR cell lines. Cells were labelled with HMFG1 (mouse mAb) and anti-mouse FITC-conjugated Ab. Dashed lines represent the negative control (PBS instead of first layer mAb)

using first layer antibodies alone or second/third layer reagents were included.

### $^{51}$Cr cytotoxicity assay

A standard $^{51}$Cr release assay was performed as previously described (Brunner et al, 1968).

***Preparation of target cells.*** Parental 410.4 and MUC1-transfected 413BCR and 425CCR culture cells at subconfluent phase were gently detached with versene–trypsine solution and single cell suspended. Viability test was carried out and groups of $10^6$ cells for each target cell line were mixed with 50 μCi of $^{51}$Cr isotonic solution (Amersham) for 1 h at 37°C in a humidified 5% carbon dioxide incubator. Subsequently, selected groups of each target cell line were then added to 200 μl of medium containing 2 μg of streptavidinylated anti-MUC1 mucin antibody construct (streptavidinylated HMFG1 IgG) and left at 4°C for 30 min. Control groups derived from each target cell line did not receive anti-MUC1 mucin antibody. Cells were then washed three times to remove unbound antibody and placed in a 96-well plate at a concentration of $5 \times 10^3$ cells per well. Maximum release was determined by wells containing control groups with added detergent (SDS) and represent 100% lysis of target cells. Spontaneous release was determined by wells containing control groups with no detergent or CTL.

***Preparation of effector cells.*** Preactivated CTL bulk cultures were harvested and viable lymphocytes were counted. Groups of $1.5 \times 10^6$ lymphocytes were resuspended in 200 μl of medium containing 2 μg of biotinylated anti-CD3 Fab fragments. Control groups did not receive biotinylated anti-CD3 Fab fragments. Cells were left at 4°C for 25 min, washed to remove excess antibody and aliquoted in 96-well plates in effector:target ratios ranging from 50:1 to 3:1 Following a 4-h incubation period, plates were spun, radioactivity of harvested supernatant was counted using an automatic gamma counter and percentage of killing was estimated using the formula: %Cytotoxicity = [(experimental release – spontaneous release) / (maximum release – spontaneous release)] × 100.

### RESULTS

### MUC1 mucin targeting

Following transfection of the 410.4 mouse mammary cell line with the human MUC1 mucin cDNA two of the resulting clones were characterized and shown to express the MUC1 mucin. Specific flow cytometric studies using the HMFG1 anti-MUC1 mucin mAb, showed that 425CCR transfectant is a moderate MUC1 mucin expressor and the 413BCR transfectant a high expressor

 © 1999 Cancer Research Campaign

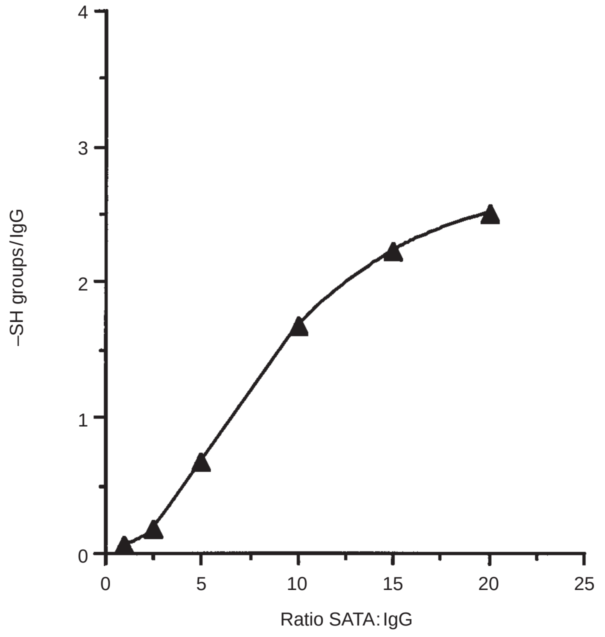

**Figure 4** Incorporation of -SH groups on HMFG1 mAb at different SATA:IgG molar ratios. The effect of the increasing concentration of SATA on the incorporation of reactive sulphydryl groups onto the HMFG1 mAb is described. Increasing molar ratios of SATA to HMFG1, shown on the abscissa, were allowed to react with 60 μM HMFG1. Sulphydryl incorporation was measured using Ellmans reagent clone (Figure 3). The HMFG1 showed stronger binding for the 413BCR cell line with 90–95% of the cells binding the mAb, while in the case of 425CCR the percentage was 60–70%. Similar to the 413BCR cell line, 95% of MCF-7-positive control cells were shown to bind the HMFG1 mAb. No antibody binding was detected in the 410.4 parental cell line or the 435DNV vector control cell line.

### Direct streptavidinylation of HMFG1

Direct streptavidinylation of HMFG1 mAb was carried out using a non-denaturing protocol (Figure 2). Initial sulphydryl group incorporation onto the HMFG1 mAb was carried out using SATA. Reaction was carried out at different SATA:HMFG1 ratios, and sulphydryl incorporation was calculated using the Ellman's assay and plotted (Figure 4). At SATA:HMFG1 ratio of 10:1 an incorporation of a mean 1.67 moles of -SH groups per mole of HMFG1 was found after deacetylation. Subsequently, a maleimide group was introduced onto the streptavidin and finally thiolated HMFG1 and maleimide-conjugated SA were mixed in equimolar ratios reacting to form an amide-thioether bond and thus streptavidinylated HMFG1 mAb. HMFG1 mAb streptavidinylated by this approach was tested for both the ability to bind MUC1 mucin-expressing cell lines and for carrying SA by flow cytometry (Figure 5). The results clearly showed that streptavidinylated HMFG1 can bind to the MUC1 mucin-expressing clone 413BCR, but not to the non-MUC1 mucin-expressing parental 410.4 cell line. Streptavidinylation of the HMFG1 mAb did not grossly affect its immunoreactivity with the 413BCR cell line which was shown to be similar to unconjugated HMFG1, with 95% of the cells showing staining. Negative control experiments carried out with the cross-linking reagents and second/third layer show no cross-reactivity with the cell membrane.

### Biotinylated anti-CD3 Fab fragments

Fab fragments of 1452C11 anti-CD3 mAb were generated by papain digestion, meticulously purified by affinity chromatography and confirmed by SDS-PAGE gel stained with Coomassie brilliant blue (data not shown) and flow cytometry (Figure 5). Flow cytometry showed 90–95% binding of purified Fab fragments to CD3-positive T-cells. Biotinylation of Fab fragments was carried out using NHS-iminobiotin at 7:1–50:1 biotin:Fab ratios. The mean number of biotin incorporation/Fab, estimated by the HABA method, was found to range from 0.2 to 8 (data not shown). A biotin:Fab ratio of 30:1 was found to result in a mean number of 3 moles of biotin incorporated per mole of Fab. Cell binding studies by flow cytometry of T-cells labelled with biotinylated anti-CD3 Fab fragments showed 90–99% positivity. In addition, the same study showed that biotin conjugated to Fab fragments retained the ability to cross-link SA.

### Cross-linking of biotinylated anti-CD3 Fab fragments to the streptavidinylated HMFG1 mAb

*Flow cytometry*

The ability of streptavidinylated HMFG1 to retain functional biotin binding site and cross-link biotinylated anti-CD3 Fab fragments was tested by flow cytometry (Figure 5). The results clearly showed cross-linking of the two antibody constructs mediated by the SA–biotin bond in 90% of the cells (Figure 5). Negative control experiments showed that no cross-reactivity occurred with the cell membrane (Figure 5).

### $^{51}Cr$ cytotoxicity assay

The biologic effect of the streptavidinylated HMFG1 mAbs and biotinylated anti-CD3 Fab fragments was tested using a $^{51}Cr$ cytotoxicity assay. Streptavidinylated HMFG1 mAb and biotinylated anti-CD3 Fab fragments were separately incubated with target and effector T-cells respectively to mimic the in vivo approach. Biotinylated anti-CD3 Fab fragments were shown to successfully redirect preactivated T-cells against target cells stained with streptavidinylated HMFG1 mAb. Lysis was achieved at approximately 70% and 40% of the high (413BCR) and the low (425CCR) MUC1 mucin expressing cell lines respectively, using an E:T ratio of 50:1 (Figure 6). The control experiments consisting of non-MUC1 mucin-expressing 410.4 parental cell line and unlabelled MUC1 mucin-transfected cells or unlabelled T-cells did not exhibit cytotoxic activity.

### DISCUSSION

We have developed a novel antibody approach for tumour immunotherapy consisting of biotinylated Fab fragments raised against the CD3 region of effector T-cells and directly streptavidinylated mAbs raised against the MUC1 mucin tumour antigen.

This is the first report describing the generation of the two antibody constructs for potential immunotherapeutic application and showing that these constructs can specifically bind the cell membrane, cross-link the CD3 receptor through a SA–biotin bond and thereby induce tumour cell killing. This novel antibody

© 1999 Cancer Research Campaign

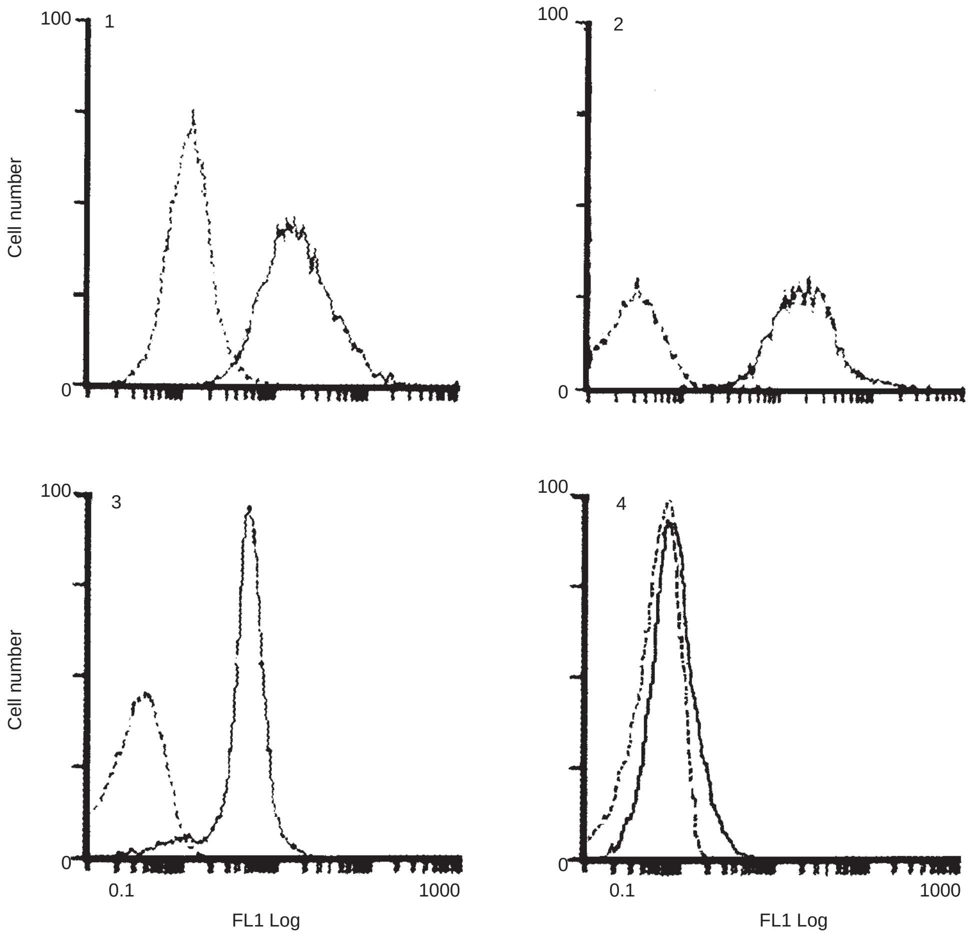

**Figure 5** Cell binding studies of the labelled Mab constructs by flow cytometry. 1: Testing of streptavidinylated HMFG1 mAb binding to 413BCR cell line. Cells were stained with streptavidinylated HMFG1, rabbit anti-streptavidin Ab and FITC anti-rabbit Ab. 2: Testing of biotinylated 1452C11 Fab fragment binding to T-cells. Cells were stained with biotinylated anti-CD3 Fab fragments and FITC-conjugated streptavidin; 95% positivity was reached. 3: Testing cross-linking of biotinylated Fab fragment to streptavidinylated HMFG1 mAb. T-cells were labelled with biotinylated Fab fragments, streptavidinylated HMFG1, rabbit anti-streptavidin Ab and anti-rabbit FITC-conjugated Ab. 4: A representative negative control experiment included T-cells labelled with biotinylated irrelevant Fab fragments, rabbit anti-streptavidin Ab and anti-rabbit FITC-conjugated Ab. Dashed lines represent the negative control (PBS instead of first layer mAb)

approach might prove advantageous over previous antibody approaches in in vivo applications as it is designed to separately label tumour cells and T-cells and induce T-cell recruitment and activation at the tumour site. A syngeneic solid tumour model composed of a breast adenocarcinoma cell line transfected with MUC1 mucin and preactivated T-cells was established to test redirection of T-cell cytotoxicity using this novel indirect antibody approach.

Streptavidinylation of HMFG1 mAb by sulphydryl-maleimide coupling (maleimide groups bound to SA react with free -SH groups on the HMFG1 antibody), resulted in a very stable amide-thioether bond without altering the mAb and SA binding properties (Hashida et al, 1984). The introduction of -SH group onto HMFG1 was achieved using SATA which offers several advantages over other methodologies such as SAMSA and SPDP (reviewed by (Duncan et al, 1983)). The most important advantage is that SATA reacts under mild and non-denaturating conditions with primary amines to form a covalently linked -SH group 'protected' from oxidation by an acetyl group. This can be 'deprotected' by hydroxylamine, which is specific for the thiol-carboxylate group, thus eliminating the potential presence of another thiol that could quench or compete with subsequent sulphydryl reactions. Our results showed 1.67 -SH groups incorporated per antibody and are in line with the results of other studies aiming at a single substituent incorporation (Duncan et al, 1983; Jones & Hudson, 1993). Duncan and colleagues found their results were in agreement with the Poisson distribution, whereby 36% of the derivatized antibody was monosubstituted, 31% bisubstituted and 24% unsubstituted.

Streptavidinylation and avidinylation of molecules has been previously achieved indirectly by conjugating biotin onto the target antibody which is then incubated with SA or avidin. The major disadvantages of this conjugation reaction are: (a) loss of at least one binding site on the SA molecule engaged in the SA–biotin bond and (b) addition of SA in the biotinylated antibody solution. In the latter case, dimeric and polymeric species consisting of several antibody molecules bound together (Hnatowich et al, 1987) may form and have to be removed prior to administration to patients (Kalofonos et al, 1990). In our study, SA was selected over avidin as it is unglycosylated and it had previously been suggested that glycans might interfere with cross-linking and increase non-specific binding to tissues (Chaiet, 1964).

 © 1999 Cancer Research Campaign

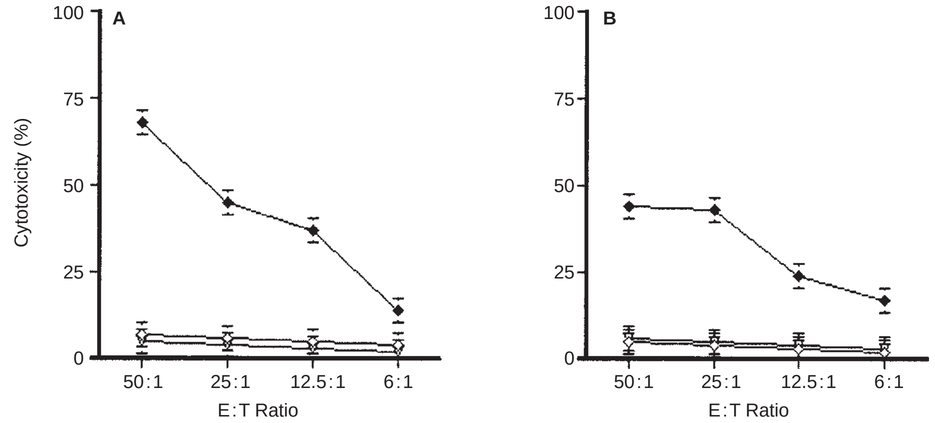

**Figure 6** $^{51}$Cr cytotoxicity assay. $^{51}$Cr labelled 413BCR, 425CCR and 410.4 tumour cell lines were incubated with/without streptavidinylated HMFG1 mAb. T-cells were incubated with/without biotinylated anti-CD3 Fab fragments. T-cells and tumour cells were incubated at different effector:target ratios and allowed to react. Data shown are representative of three independent experiments. Bars indicate standard error. At all E:T ratios the percentage lysis of MUC1 expressors in the presence of both antibody constructs differs significantly from the controls. **(A)** (◆) 413BCR cells stained with streptavidinylated HMFG1 were incubated with T-cells stained with biotinylated 1452C11 Fab fragments; (◇) unstained 413BCR cells were incubated with stained T-cells; (∇) stained 413BCR cells were incubated with unstained T-cells. **(B)** (◆) 425CCR cells stained with streptavidinylated HMFG1 were incubated with T-cells stained with biotinylated 1452C11 Fab fragments; (◇) unstained 425CCR cells were incubated with stained T-cells; (∇) 410.4 parental cell line pre-incubated with streptavidinylated HMFG1 in the presence of biotinylated 1452C11 Fab fragment-labelled T-cells

In a review of the current literature only a single study could be found reporting direct IgG conjugation to SA by chemical methods for immunoscintigraphy purposes (Sheldon, 1992). In another study, direct conjugation of SA to a single-chain antibody fragment (Dubel, 1995) was achieved by DNA recombinant methods. The major disadvantage of the later approach is that laborious purification procedures of scFv-SA from bacteria lysates are required since the amount of scFv recovered per litre of bacterial culture supernatant is very low (100–500 μg) (George et al, 1994).

The current study also reports the refinement of procedures for the chemical generation of biotinylated anti-CD3 Fab fragments. The biotin:Fab ratio of 30:1 was selected as at this ratio the mean number of biotin moles incorporated per Fab is three (the majority of Fab fragments have incorporated 2–4 biotin molecules) while antigen-binding specificity is retained. Furthermore, a previous study carried out on NHS-esters, similar to the biotin ester used in our study, reported hydrolysis reactions to occur (Anjaneyula et al, 1987) making a higher of 1 biotin:Fab ratio desirable. Reports on biotinylation of Fab fragments are present in the literature, including a recent study by Otsuji describing the biotinylation of genetically engineered Fab fragments, using NHS biotin (Otsuji, 1996) and a report by Weiss describing the complete generation of biotinylated Fab fragments by recombinant methods (Weiss, 1994).

Control cell binding studies showed that the conditions used in the coupling reactions of both the biotinylation of Fab fragments and of the HMFG1 streptavidinylation did not affect the immunoreactivity of the conjugates nor the ability of SA to bind biotin.

In our in vitro studies, MUC1 mucin-positive tumour cells labelled with streptavidinylated HMFG1 provided a matrix for multimeric binding of the biotinylated Fab fragments and facilitated CD3 cross-linking on preactivated T-cells resulting in tumour cell killing. Up to date the use of preactivated T-cells in a BiAb setting remains controversial. Current opinion indicates that Ab-mediated cross-linking of the CD3 complex is not in itself sufficient to activate resting T-cells. Many studies have shown that primed or preactivated T-cell use is a prerequisite for successful BiAb application (e.g. Weiner, 1994). Methods of in vivo and ex-vivo T-cell activation have been developed using mainly cytokines such as IL-2 and mitogens such as ConA and staphylococcal enterotoxin-B (Weiner et al, 1994). Although the signalling pathways by which IL-2 mediates its effect are not fully understood it is well-established in vitro that IL-2 is a potent T-cell mitogen. A recently developed clonogenic assay showed the importance of repeated administration of BiAbs and IL-2 for tumour killing (Haagen et al, 1995). Redirection of T-cells was shown to occur in the presence of cells expressing MUC1 mucin (413BCR and 425CCR) that could engage the streptavidinylated HMFG1 arm but not in the presence of cells not expressing the target antigen (410.4). Induction of tumour cell lysis was strictly dependent upon the expression of the MUC1 mucin molecule on target cells as demonstrated by the different susceptibility to lysis of differentially expressing MUC1 mucin-transfected cells and the negative parental cell line.

The results of the in vitro cytotoxicity assays testing the novel SA–biotin indirect antibody approach are in agreement with other studies testing redirection of T-cells against tumour cells with BiAb and previous indirect methods (Perez et al, 1985; George et al, 1994).

The mechanisms through which antibody redirected T-cells exert their cytotoxic effect over antigen expressing tumour cells have not been fully defined. A recent study showed that anti-CD3 redirection of T-cells induced killing of $BCL_1$ expressing lymphoma via the FAS pathway suggesting the process is mediated by apoptosis (Armstrong & Vitetta, 1996). Interestingly, another in vitro study carried out by Gimmi and colleagues, investigating the paucity of clinical responses in patients with immune responses to MUC1 mucin expressing malignancies, showed that MUC1 mucin was responsible for inducing apoptosis of activated T-cells (Gimmi, 1996). Using our antibody mediated T-cell redirecting approach such a tumour escape mechanism could be overridden. Our theory is supported by the findings of Katayose et al (1996) who showed T-cell redirection against MUC1 mucin

*© 1999 Cancer Research Campaign* 

expressing cells by anti-CD3 × anti-MUC1 mucin BiAb results in tumour cell lysis.

When compared to BiAb methodologies our SA–biotin indirect approach has similar advantages to the indirect approaches developed by Gilliland et al (1988) and George et al (1994). Specifically, it has the advantage of being less expensive, since the biotinylated anti-CD3 Fab fragments can be used as a 'universal arm' to redirect effector cells against a variety of tumours, and furthermore offers the opportunity of targeting more than one tumour associated antigen at the same time. When compared to the previous indirect antibody approaches (Gilliland et al, 1988; George et al, 1994), the use of streptavidinylated anti-tumour antibodies and biotinylated anti-CD3 Fab fragments for effector cell recruitment could provide, at least theoretically, two additional advantages. First, the SA–biotin bonding is based on a stronger interaction than antibody–antibody. Specifically, while the association constant ($K_a$) of the antibodies for their antigen varies from $10^5$–$10^{12}$ $M^{-1}$ (Morris, 1995) the SA–biotin bonding is one of the strongest non-covalent interactions with $K_a = 10^{15}$ $M^{-1}$ (Green, 1975). Such strong $K_a$ may be an advantage in an in vivo model where there is a rapid turnover of tumour-infiltrating lymphocytes. Secondly, each SA molecule can bind four molecules of biotin (Argarana, 1986). As a result, in an in vivo model a streptavidinylated mAb to biotinylated Fab ratio of four could theoretically be translated to an improved effector to tumour cell ratio. Therefore, sequential administration of streptavidinylated mAb followed by T-cells labelled with biotinylated anti-CD3 (provided that high tumour:background ratio is achieved), should ensure that most tumour cells would be coated with streptavidinylated Fab fragments and that T-cells would only be activated by the SA at the surface of the target cells and not by residual unbound streptavidinylated mAb.

More importantly it is possible that conventional BiAb might diffuse directly into the interstitial space of the tumour. However, it seems more likely that the BiAb would first bind to the effector cell, extravasate and then be carried into the tumour tissue. As a result, smaller antibody constructs would have the advantage over conventional BiAbs in penetrance of solid tumours. Every biotinylated Fab fragment labelled CTL that has entered the tumour tissue could become cross-linked on the streptavidinylated mAb bound on tumour cells and react against the tumour cells, regardless of its specificity. Furthermore, antigen specificity of labelled CTL would be blocked by the anti-CD3 Fab monomers resulting only in activation of the cell at the tumour site where biotinylated Fab fragments become cross-linked (Perez et al, 1985) without inappropriate cross-linking occurring elsewhere.

In summary, we developed a novel indirect antibody approach for immunotherapy of solid tumours based on the separate labelling of tumour cells (MUC1 mucin) and T-cells (CD3 epitope) with antibodies chemically conjugated to biotin and streptavidin moieties that bring them into proximity resulting in tumour cell death. Our future studies aim to investigate whether the separate (thus ex-vivo) labelling of T-cells with biotinylated Fab fragments and tumour cells with streptavidinylated HMFG1 will result in T-cell recruitment at the tumour site and CTL-mediated lysis of MUC1 mucin-transfected BALB/c tumour cell lines in a syngeneic mouse model. If successful, this approach could open new avenues for selective destruction of human tumours by activated T-cells avoiding the complications associated with the currently available treatments.

### ACKNOWLEDGEMENTS

The authors are grateful to Dr Andrew George for critical review of the manuscript. This study was sponsored by a LOCR8 grant, the Byggott studentship, the Greek Orthodox Charity and Efi Nomikos donation.

### REFERENCES

Anjaneyula PSR and Staros JV (1987) Reactions of N-hydroxysuccinimide active esters. *Int J Pep Pro Res* **30**: 117–124

Batra SK, Kern HF, Worlock AJ, Metzgar RS and Hollingsworth MA (1991) Transfection of the human Muc 1 mucin gene into a poorly differentiated human pancreatic tumor cell line, Panc1: integration, expression and ultrastructural changes. *J Cell Sci* **100**: 841–849

Blazar BA, Laing CA, Miller FR and Heppner GH (1980) Activity of lymphoid cells separated from mammary tumors in blastogenesis and Winn assays. *J Natl Cancer Inst* **65**: 405–410

Brunner KT, Mauel J, Cerottini J, Chapuis B (1968) Quantitative assay of the lytic action of immune lymphoid cells on 51-Cr-labelled allogeneic target cells in vitro; inhibition by isoantibody and by drugs. *Immunology* **14**: 181–196

Demanet C, Brissinck J, Moser M, Leo O and Thielemans K (1992) Bispecific antibody therapy of two murine B-cell lymphomas. *Int J Cancer Suppl* **7**: 67–68

Devine PL and McKenzie IF (1992) Mucins: structure, function, and associations with malignancy. *Bioessays* **14**: 619–625

Duncan RJ, Weston PD and Wrigglesworth R (1983) A new reagent which may be used to introduce sulfhydryl groups into proteins, and its use in the preparation of conjugates for immunoassay. *Anal Biochem* **132**: 68–73

Ellman G (1959) Tissue sulfhydryl groups. *Arch Biochem Biophys* **82**: 70–77

Epenetos AA, Britton KE, Mather S, Shepherd J, Granowska M, Taylor Papadimitriou J, Nimmon CC, Durbin H, Hawkins LR, Malpas JS and Bodmer WF (1982) Targeting of iodine-123-labelled tumour-associated monoclonal antibodies to ovarian, breast, and gastrointestinal tumours. *Lancet* **2**: 999–1005

George AJ, Titus JA, Jost CR, Kurucz I, Perez P, Andrew SM, Nicholls PJ, Huston JS and Segal DM (1994) Redirection of T cell-mediated cytotoxicity by a recombinant single-chain Fv molecule. *J Immunol* **152**: 1802–1811

Gilliland LK, Clark MR and Waldmann H (1988) Universal bispecific antibody for targeting tumor cells for destruction by cytotoxic T cells. *Proc Natl Acad Sci USA* **85**: 7719–7723

Green NM (1965) A spectrophotometric assay for avidin and biotin based on binding dyes by avidin. *Biochem J* **94**: 23c–24c

Green NM (1975) Avidin. *Adv Protein Chem* **29**: 85–133

Haagen IA, Geerars AJ, de Lau WB, Bast BJ and de Gast BC (1995) The efficacy of CD3 × CD19 bispecific monoclonal antibody (BsAb) in a clonogenic assay: the effect of repeated addition of BsAb and interleukin-2. *Blood* **85**: 3208–3212

Hashida S, Imagawa M, Inoue S, Ruan KH and Ishikawa E (1984) More useful maleimide compounds for the conjugation of Fab′ to horseradish peroxidase through thiol groups in the hinge. *J Appl Biochem* **6**: 56–63

Havran WL and Allison JP (1988) Developmentally ordered appearance of thymocytes expressing different T-cell antigen receptors. *Nature* **335**: 443–445

Heppner GH, Dexter DL, DeNucci T, Miller FR and Calabresi P (1978) Heterogeneity in drug sensitivity among tumor cell subpopulations of a single mammary tumor. *Cancer Res* **38**: 3758–3763

Hnatowich DJ, Virzi F and Rusckowski M (1987) Investigations of avidin and biotin for imaging applications. *J Nucl Med* **28**: 1294–1302

Kalofonos HP, Rusckowski M, Siebecker DA, Sivolapenko GB, Snook D, Lavender JP, Epenetos AA and Hnatowich DJ (1990) Imaging of tumor in patients with indium-111-labeled biotin and streptavidin-conjugated antibodies: preliminary communication [see comments]. *J Nucl Med* **31**: 1791–1796

Laemmli UK, Beguin F and Gujer Kellenberger G (1970) A factor preventing the major head protein of bacteriophage T4 from random aggregation. *J Mol Biol* **47**: 69–85

Le Doussal JM, Barbet J and Delaage M (1992) Bispecific-antibody-mediated targeting of radiolabeled bivalent haptens: theoretical, experimental and clinical results. *Int J Cancer Suppl* **7**: 58–62

Leo O, Foo M, Sachs DH, Samelson LE and Bluestone JA (1987) Identification of a monoclonal antibody specific for a murine T3 polypeptide. *Proc Natl Acad Sci USA* **84**: 1374–1378

Miller BE, Miller FR, Machemer T and Heppner GH (1993) Melphalan sensitivity as a function of progressive metastatic growth in two subpopulations of a mouse mammary tumour. *Br J Cancer* **68**: 18–25

© 1999 Cancer Research Campaign

Nicoletti I, Migliorati G, Pagliacci MC, Grignani F and Riccardi C (1991) A rapid and simple method for measuring thymocyte apoptosis by propidium iodide staining and flow cytometry. *J Immunol Methods* **139**: 271–279

Nitta T, Sato K, Yagita H, Okumura K and Ishii S (1990) Preliminary trial of specific targeting therapy against malignant glioma. *Lancet* **335**: 368–371

Paganelli G, Riva P, Deleide G, Clivio A, Chiolerio F, Scassellati GA, Malcovati M and Siccardi AG (1988) In vivo labelling of biotinylated monoclonal antibodies by radioactive avidin: a strategy to increase tumor radiolocalization. *Int J Cancer Suppl* **2**: 121–125

Perez P, Hoffman RW, Shaw S, Bluestone JA and Segal DM (1985) Specific targeting of cytotoxic T cells by anti-T3 linked to anti-target cell antibody. *Nature* **316**: 354–356

Portoles P, Rojo J, Golby A, Bonneville M, Gromkowski S, Greenbaum L, Janeway CJ, Murphy DB and Bottomly K (1989) Monoclonal antibodies to murine CD3 epsilon define distinct epitopes, one of which may interact with CD4 during T cell activation. *J Immunol* **142**: 4169–4175

Soule HD, Vazguez J, Long A, Albert S and Brennan M (1973) A human cell line from a pleural effusion derived from a breast carcinoma. *J Natl Cancer Inst* **51**: 1409–1416

Staerz UD, Kanagawa O and Bevan MJ (1985) Hybrid antibodies can target sites for attack by T cells. *Nature* **314**: 628–631

Taylor Papadimitriou J and Epenetos AA (1994) Exploiting altered glycosylation patterns in cancer: progress and challenges in diagnosis and therapy. *Trends Biotechnol* **12**: 227–233

Weiner GJ, Kostelny SA, Hillstrom JR, Cole MS, Link BK, Wang SL and Tso JY (1994) The role of T cell activation in anti-CD3 × antitumor bispecific antibody therapy. *J Immunol* **152**: 2385–2392

Zhu H, Jain RK and Baxter LT (1998) Tumor pretargeting for radioimmunodetection and radioimmunotherapy. *J Nucl Med* **39**: 65–76

*© 1999 Cancer Research Campaign*
